# Depicting the Core Transcriptome Modulating Multiple Abiotic Stresses Responses in Sesame (*Sesamum indicum* L.)

**DOI:** 10.3390/ijms20163930

**Published:** 2019-08-13

**Authors:** Komivi Dossa, Marie A. Mmadi, Rong Zhou, Tianyuan Zhang, Ruqi Su, Yujuan Zhang, Linhai Wang, Jun You, Xiurong Zhang

**Affiliations:** 1 Oil Crops Research Institute of the Chinese Academy of Agricultural Sciences, Key Laboratory of Biology and Genetic Improvement of Oil Crops, Ministry of Agriculture, Wuhan 430062, China; 2 State Key Laboratory of Agricultural Microbiology, Huazhong Agricultural University, Wuhan 430070, China

**Keywords:** stress marker genes, sesame, gene co-expression, abiotic stress tolerance, hub genes, meta-analysis

## Abstract

Sesame is a source of a healthy vegetable oil, attracting a growing interest worldwide. Abiotic stresses have devastating effects on sesame yield; hence, studies have been performed to understand sesame molecular responses to abiotic stresses, but the core abiotic stress-responsive genes (CARG) that the plant reuses in response to an array of environmental stresses are unknown. We performed a meta-analysis of 72 RNA-Seq datasets from drought, waterlogging, salt and osmotic stresses and identified 543 genes constantly and differentially expressed in response to all stresses, representing the sesame CARG. Weighted gene co-expression network analysis of the CARG revealed three functional modules controlled by key transcription factors. Except for salt stress, the modules were positively correlated with the abiotic stresses. Network topology of the modules showed several hub genes predicted to play prominent functions. As proof of concept, we generated over-expressing Arabidopsis lines with hub and non-hub genes. Transgenic plants performed better under drought, waterlogging, and osmotic stresses than the wild-type plants but did not tolerate the salt treatment. As expected, the hub gene was significantly more potent than the non-hub gene. Overall, we discovered several novel candidate genes, which will fuel investigations on plant responses to multiple abiotic stresses.

## 1. Introduction

Climate change causes the rising of sea levels, a decrease of available land for farming, and increased frequencies of severe droughts, intense precipitation events, elevated temperatures, as well as salt and heavy metals contamination of soils. Crop productivity and survival is tightly linked to its environment, which is being altered due to climate change [1], impairing crop yields and leading to enhanced risks of famine worldwide [2]. Being a sessile organism, plants have evolved an enormous capacity to adapt to environmental changes including heat, drought, salinity, osmotic pressure, waterlogging, etc., by modulating their physiology, growth and development. Until recently, numerous researches have focused on plant responses to individual abiotic stress [3,4,5,6,7,8]. Although these studies have potential applications in crop improvement for abiotic stress tolerance, crops growing in natural habitats are often exposed to multiple environmental stresses occurring simultaneously or at different development stages, which inflicts a more severe reduction in yields as compared to a single stress [9,10]. Hence, development of crops able to tolerate a wide range of abiotic stresses with high productivity is imperative in order to meet various socio-economic and agro-economic challenges in the current climate change scenario [11,12]. However, the interactive improvement of multiple abiotic stress tolerances is a challenge [13], since increasing tolerance to one stress may be at the expense of tolerance to another [9,14]. Therefore, there is a need to investigate the metabolic pathways and regulatory networks of multiple abiotic stress acclimations in plants and obtain candidate genes for manipulation to improve tolerance to multiple abiotic stresses. 

In recent years, it has become evident that a common battery of responses can be triggered by various stresses. For example in yeasts, it has been discovered that a set of genes are constantly activated upon exposure to various abiotic stresses and represent the core environmental stress responsive genes [15,16,17,18,19]. Similarly, the core environmental stress responsive genes were unraveled in plants, first in *Arabidopsis thaliana* through the AtGenExpress abiotic stress experiment [20,21,22] and later confirmed in various plant species, such as rice, soybean, banana, *Brachypodium distachyon* or barley [13,23,24,25,26,27], indicating the conservation of a core genome modulating various abiotic stresses responses between species. Thus, it is critical to compare and analyze different kinds of abiotic stress responses to find the common genes and understand how they regulate plant’s adaptations to the multiple environmental stresses. This information will guide in strategies to enhance crop tolerance to multiple abiotic stresses.

Consumers become more and more health conscious, with a sharper focus on health-promoting diets. Oils and fats are an important part of a balanced and healthy diet. Among the most nutritious and healthy vegetable oils, sesame (*Sesamum indicum* L.) oil occupies a pro-eminent position because of the low level of saturated fatty acids (less than 15%) and the presence of strong antioxidants, reported to have health-promoting effects such as lowering cholesterol levels and hypertension [28,29], neuroprotective effects against hypoxia or brain damage [30] and reducing the incidence of certain cancers [31,32]. Therefore, demands for and world trade in sesame seeds have increased rapidly during the last two decades [33]. Although the global sesame planting area is extending, particularly in Africa, the productivity and yield are still very low, resulting in a huge gap between seed demand and supply [34]. In fact, sesame is cultivated in harsh environments and its growth and development are greatly affected by the adverse conditions [35]. Drought, waterlogging, heat and salt stresses represent the leading abiotic factors impairing sesame yield and productivity [36] and several of these environmental stressors often occur in combination at different growth stages. For example, in the arid and semi-arid areas of Africa, America and Asia, extreme heat and drought stresses co-occur and challenge sesame crop. In East and South Asia, waterlogging and salinity stress devastate sesame field. Over the past five years, tremendous efforts have been made to decipher the molecular basis of abiotic stress response and tolerance in sesame [6,7,8,37,38]. However, none of these studies has performed a meta-analysis of diverse transcriptome data to elucidate the similarities and differences among stress response pathways, and importantly, decode the core abiotic stress-responsive genes (CARG) in sesame. 

Within the CARG, understanding how specific transcriptional changes are linked to stress adaptations and identifying central hubs controlling this interaction remain the main challenge. Weighted Gene Co-expression Network Analysis (WGCNA) is one of the widely used computational tools to detect co-expression modules in transcriptome data and identify hub genes playing preponderant functions based on a network construction [39]. Using WGCNA analysis of the RNA-Seq data, Shahan et al. [40] identified key regulators of flower and fruit development in strawberry. WGCNA has also been utilized to detect coexpression modules and major players for multiple biotic and abiotic stresses responses in various plant species, including Arabidopsis, rice, maize, soybean and poplar [41,42,43,44,45].

In this study, we conducted a meta-analysis of 72 RNA-Seq data from drought, salt, osmotic and waterlogging stresses aiming at identifying the CARGs modulating sesame responses to multiple abiotic stresses. By applying WGCNA, we revealed for the first time the co-expressed functional modules within these CARGs and highlighted the major genes to target for sesame improvement towards tolerance to multiple abiotic stresses.

## 2. Results

### 2.1. Overview of the Transcriptome Data and Gene Expression Profile under Various Stress Treatments

In this study, we analyzed the global gene expression profiles of sesame under various abiotic stress treatments based on the RNA-Sequencing (RNA-Seq) technology. In total, four datasets from salt stress [8], drought stress [7], waterlogging stress [6] and a newly generated RNA-Seq data under osmotic stress, comprising of 30, 24, 6 and 12 samples, respectively, were investigated (Table 1). In the selected studies, stress-sensitive and tolerant varieties were used. Additionally, all RNA-Seq datasets had three replicates per treatment, untreated controls for each treatment, and the tissue type was primarily the root. After cleaning and filtering the RNA-Seq datasets, we obtained clean reads ranging from 13.5 to 230 Gb with a total of 25,319 uniquely expressed genes among the 72 samples (Table 1).

### 2.2. Identification of DEGs, Core Conserved DEGs in Response to Abiotic Stress

**Figure 1:**
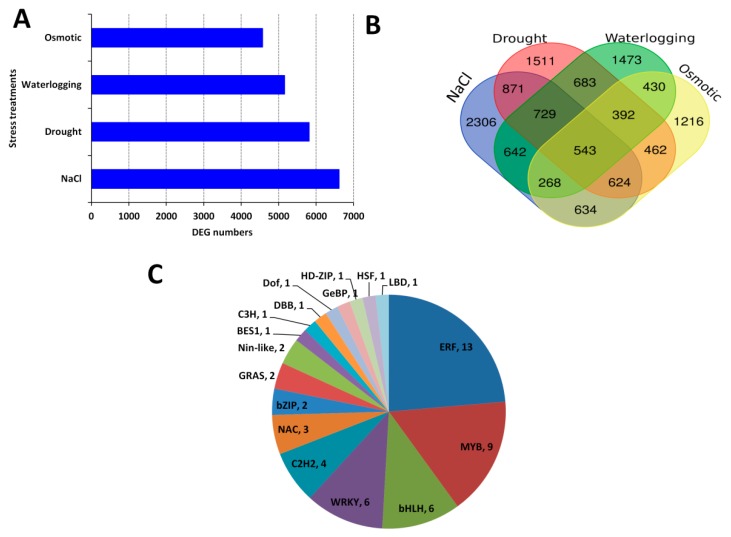
Identification of the core abiotic stress responsive genes (CARG) in sesame. (**A**) Differentially expressed genes detected between control and stress treatments. (**B**) Venn Diagram showing stress specific genes and CARGs. (**C**) Major transcription families enriched in the sesame CARGs.

For each dataset, we compared the gene expression under control condition to the stress condition in order to identify the differentially expressed genes (DEGs). We identified more DEGs under salt stress treatment compared to the other stress treatments, indicating that salt stress triggers the most intense gene regulation in sesame (Figure 1A). A total of 12,784 DEGs were identified among different samples in the datasets. We cross-compared the identified DEGs among the four datasets aiming at identifying the core conserved DEGs in response to all the four abiotic stresses. The result showed that large numbers of DEGs were stress-specific; however, 543 genes constantly participate in sesame responses to its major abiotic stresses and represent the core abiotic stress responsive genes (CARG) (Figure 1B; Table S2; Figure S1). To confirm these CARGs, we selected 10 independent sesame genotypes and evaluated the expression fold change (FC) of 50 randomly selected genes within the CARGs under drought, salt, osmotic, heat and waterlogging stresses compared to the control. As shown in Figure S2, the expression levels of all the tested genes were highly altered under stress (|FC|>2), showing that the proposed CARGs are well conserved in sesame and may be functional under a more diverse arrays of abiotic stresses. 

We further searched for the enriched transcription factor (TF) families within these CARGs. As shown in Figure 1C, 18 TF families were represented but the ERF, MYB, bHLH and WRKY were overrepresented, denoting that these TF families are the major regulators of sesame responses to multiple abiotic stresses.

### 2.3. WGCNA and Detection of Functional Modules 

**Figure 2:**
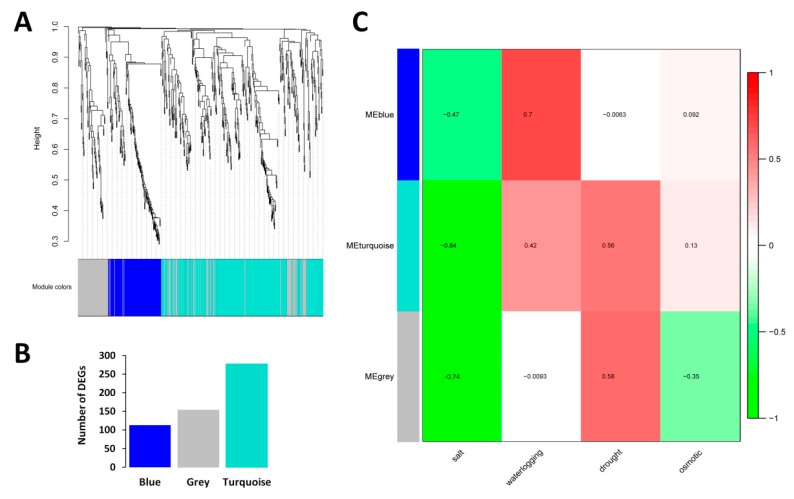
Detection of co-expressed modules in the sesame core abiotic stress responsive genes based on WGCNA. (**A**) Dendrogram showing the different genes clustered into co-expressed modules. (**B**) Number of assigned DEGs to the different modules. (**C**) Association between co-expressed modules and abiotic stresses in sesame. The numbers represent the Pearson correlation coefficients. Positive correlation is colored in red while negative correlation is colored in green.

Weighted gene co-expression network analysis (WGCNA) was conducted on the CARGs to reveal the different modules of co-expressed genes. WGCNA divided the 543 core DEGs into three different modules named as Blue, Turquoise and Grey, containing 113, 276 and 154, genes respectively (Figure 2A,B; Table S3). Association of the detected modules and the abiotic stresses indexes showed that all the three modules respond differently to the abiotic stresses, except for salt stress. In fact, all the three modules were negatively correlated with salt stress (r = −0.47, −0.84, −0.74 for Blue, Turquoise and Grey, respectively), suggesting that the CARGs should be down-regulated to allow sesame survive under salt stress (Figure 2C). In addition, with all modules taken together, we could observe that the CARGs engage distinct responses according to the stress, highlighting the key roles played by the regulator genes to shape the stress-specific responses. 

To explore the particular biological processes involving the three modules detected by WGCNA, we performed GO enrichment analysis. Blue module related genes represent the basal defense of sesame as evidenced by the enriched GO terms such as ‘defense response’ and ‘response to biotic stimulus’ (Figure S3A). Grey module related genes were enriched in the transporter activity role (Figure S3B). Finally, in Turquoise module, ‘iron ion binding’ and ‘heme binding’ were detected as the most enriched GO terms, which are well known to be involved in abiotic stress responses in plants [46,47] (Figure S3C). These results further support the premise that the co-expressed gene modules of the sesame CARGs play different functions in response to abiotic stresses.

### 2.4. Networks Displaying Relationships among Genes within Co-Expressed Modules

**Figure 3:**
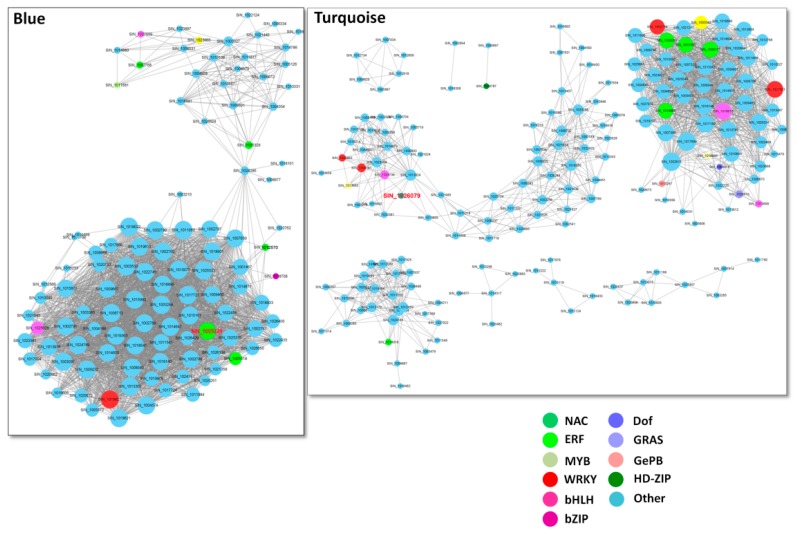
Co-expressed network analysis of Blue module and Turquoise module. The size of node circle is positively correlated with the number of the interacting gene partners. The gene names marked in red are those selected for validation using transgenic Arabidopsis approach.

To understand the gene interaction within each module, we constructed the gene network using the Cytoscape software. Genes in the different modules were subsequently divided into different clusters, each having network of different number of genes (Figure 3). TFs are represented with different node colors except sky blue and the size of node circle is positively correlated with the number of genes it interacts (Figure 3). Genes with biggest node sizes represent the hub genes. In Blue module, we observed two clusters of genes connected by the gene SIN_1024285 (transmembrane protein 45B-like). We identified several hub genes, including SIN_1003530 (2-aminoethanethiol dioxygenase), SIN_1011060 (pyruvate decarboxylase 1), SIN_1018022 (pentatricopeptide repeat-containing protein), SIN_1003097 (NA), SIN_1003294 (NA), SIN_1006713 (cationic amino acid transporter 1-like), SIN_1013309 (alcohol dehydrogenase 3), SIN_1014647 (squalene monooxygenase), SIN_1017721 (NA), SIN_1024789 (ATP-dependent 6-phosphofructokinase) etc. Furthermore, some key TFs were also present SIN_1005329 (ERF) and SIN_1019627 (WRKY), which may play important regulation role in this module (Figure 3). Turquoise module contains complex clusters of genes, implying diverse biological functions within this module (Figure 3). The key hub genes detected are SIN_1017899 (NA), SIN_1002615 (G-type lectin S-receptor-like serine/threonine-protein kinase), SIN_1005524 (exocyst complex component EXO70A1-like), SIN_1017189 (NA), SIN_1007389 (NA), SIN_1016615 (NA), SIN_1009465 (pectinesterase 1-like), SIN_1021953 (WRKY) and SIN_1013789 (MAPK). In the Grey module, one main cluster surrounded by several small clusters of genes was detected (Figure S4). Several hub genes, including SIN_1002211 (aspartic proteinase nepenthesin-1), SIN_1003190 (protein ECERIFERUM 1), SIN_1019848 (GDSL esterase), SIN_1008670 (18.2 kDa class I heat shock protein), SIN_1014764 (3-ketoacyl-CoA synthase 4) and SIN_1004965 (glucan endo-1,3-β-glucosidase 13), may play preponderant roles in this module.

**Figure 4:**
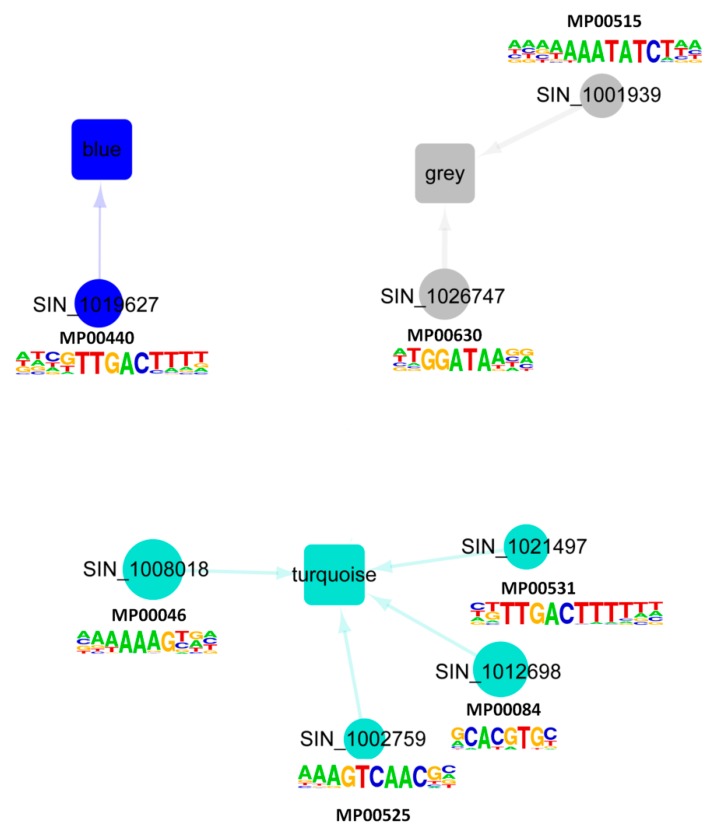
Predicted directional interactions of TFs and the co-expressed modules in the sesame CARGs. Network plots of inferred connections between TF and genes in the three modules. The promoter sequences of genes associated with each module were tested for overrepresentation of DNA motifs shown to be bound to TFs that are differentially transcribed following stress treatments. Each TF with a known motif is represented by a colored circle corresponding to its appertaining module. The different modules are represented by a rectangle. An edge between a TF and a module indicates significant enrichment of the corresponding binding motif in that module. The size of each TF node is proportional to the number of predicted regulated downstream genes. Logos of the seven enriched DNA binding motifs within the promoter regions of the genes belonging to each module detected by WGCNA were added.

Next, we extended our analysis to unveil the major regulators of the different co-expressed gene modules of the CARGs in sesame. First, we screened for overrepresented regulatory motifs in the 1 kb promoter regions of genes within each module. Seven TF binding motifs were enriched in the analyzed promoter regions (Table S4). Then, we constructed the gene regulatory networks predicting directional interactions between CARG regulators and targets associated with the three modules using the TF DNA binding motif information. Figure 4 presents the generated regulatory networks, in which the circular nodes represent the key regulators connected by an edge to a module. The size of the nodes is proportional to the number of the inferred regulated genes harboring the corresponding TF binding motifs in the promoter region and the nodes are colored according to the appertaining module. Our predicted networks showed the main TFs regulating gene expression within each module. An intense transcriptional activity was predicted in the Turquoise module, having the highest number of predicted regulators. The networks also highlighted the master players (SIN_1026747 (MYB), SIN_1012698 (bHLH) and SIN_1008018 (Dof)), which are predicted to regulate significant numbers of target genes in their modules and represent potential genes to exploit for the enhancement of sesame tolerance to various abiotic stresses. We infer that these regulators are the key genes that shape the sesame CARG specific responses to the major abiotic stresses.

### 2.5. Validation of Hub and Non-Hub TFs from the Co-Expressed Modules of the Sesame CARGs in Transgenic Arabidopsis

We selected two transcription factor encoding genes: SIN_1005329 (SiERF5) and SIN _1026079 (SiNAC104) to confirm their involvement in various abiotic stress responses using Arabidopsis system. In fact, sesame resilience to the genetic manipulation is still significant enough to justify the use of a heterologous system such as *Arabidopsis thaliana*. SiERF5 is a hub gene from Blue module while SiNAC104 is a non-hub gene from Turquoise module (Figure 3) and both were induced at different time points after stress treatments (Table S2). We hypothesized that the over-expression of SiERF5 will confer higher abiotic stress tolerance than the over-expression of SiNAC104 in Arabidopsis, given their contrasting importance in their respective modules.

**Figure 5:**
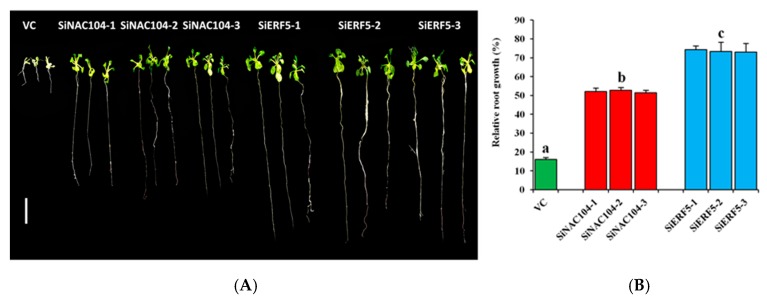
Functional characterization of SiERF5- and SiNAC104-overexpressing lines and their counterparts vector control (VC) plants under osmotic stress induced by 250 mM Mannitol addition to the MS medium. (**A**) Phenotypes of the transgenic and VC plants under stress. The bar represents 1 cm (**B**) relative root length estimated as the ratio of the root length recorded after 10 days under stressed and control MS mediums. Values are means ±SD from two independent measurements. Bars with different letters are significantly different (*p* ≤ 0.05).

**Figure 6:**
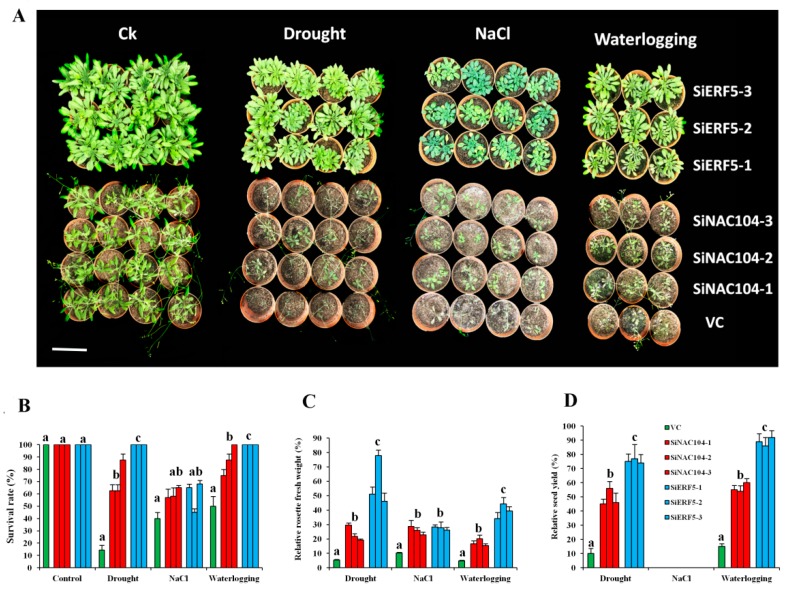
Functional characterization of SiERF5- and SiNAC104-overexpressing lines and their counterparts vector control (VC) plants under drought (20 days), salt (200 mM NaCl) and waterlogging (18 days). (**A**) Phenotypes of the plants. The bar represents 8 cm (**B**) Survival rate of the plants. (**C**) Relative rosette fresh weight. (**D**) Relative seed yield. The relative values are estimated as the ratio of the data recorded after stress period in stressed and control plants. Values are means ± SD from two independent measurements. For each treatment, bars with different letters are significantly different (*p* ≤ 0.05).

We generated several T3 homozygous over-expressing Arabidopsis lines for both genes, of which three independent lines for each gene (SiERF5-1, SiERF5-2, SiERF5-3 and SiNAC104-1, SiNAC104-2, SiNAC104-3) were selected for stress applications. Overexpression of SiERF05 increased the leaf biomass which may result from a pleiotropic effect as compared to SiNAC104-overexpressing plants and the vector control (VC) plants (Figure S5). qRT-PCR was used to confirm the integration and the expression of the transgene (Figure S5). Under osmotic stress induced by 250 mM Mannitol addition to the MS medium, the growth performance of all the transgenic lines was significantly better than the VC plants (*p* < 0.001), indicating that the transgenes confer osmotic stress tolerance (Figure 5A,B). Furthermore, we observed that the SiERF05-overexpressing lines maintained significantly better relative root growth than the SiNAC104-overexpressing lines (Figure 5A,B). Next, we evaluated the performance of the overexpressing lines and VC plants under drought (20 days), salinity (200 mM NaCl) and waterlogging (18 days). As shown in Figure 6A, SiERF5 overexpression had pleiotropic effect including delaying of flowering time and increase of the rosette biomass compared to the SiNAC104-overexpressing lines and VC plants, showing that SiERF5 participates in plant growth and development. This result hints that Blue and Turquoise modules related genes are functionally different, as demonstrated by the GO enrichment analysis. Under stress treatments, all the plants were affected, reflected by the reduced biomass (Figure 6A). However, similar to the osmotic stress treatment, the transgenic lines significantly better sustained drought, salt and waterlogging stresses than the VC plants as evidenced by their higher survival rate, relative rosette fresh weight and relative seed yield (Figure 6B–D). In addition, the results indicated that the two transgenes were more potent under drought and waterlogging stresses compared to the salt stress since we had a more pronounced biomass reduction and no seed yield under salt. Furthermore, we observed a significantly higher tolerance to the different abiotic stresses by SiERF5-overexpressing lines than the SiNAC104-overexpressing lines. Overall, these findings support the argument that the proposed sesame CARGs are functionally active under various abiotic stresses. In addition, this finding supports our formulated hypothesis, denoting that the position of a gene in the co-expressed modules (hub genes or non-hub genes) may reflect the importance of its function, which will guide the choice of high potential genes from the sesame CARGs for germplasm enhancement.

## 3. Discussion

Reinforcing crop’s tolerance to abiotic stress has become a hot issue in the current scenario of climate change, which is boosting extreme weathers, posing a growing threat to sustainable agriculture. Because of the multitude of environmental stresses affecting crop survival and productivity in the field, the identification of potential genes conferring tolerance to multiple abiotic stresses is highly desirable [9,48]. In this study, we employed various abiotic stresses RNA-Seq datasets (waterlogging, drought, salt and osmotic stresses) from diverse sesame genotypes with contrasting levels of tolerance. Our meta-analysis unveiled 543 genes as the core abiotic stress responsive genes (CARG) modulating sesame responses to multiple abiotic stresses. We validated a subset of these CARGs in ten independent sesame genotypes, showing that these CARGs are not genotype-dependent but are well conserved in the sesame species. The alteration of the expression levels of selected CARGs under heat stress further hints that the proposed CARGs may be functional under a more diverse set of environmental stressors. Nonetheless, our studied transcriptome data were mainly from sesame root samples; hence, additional analyses are needed to check how the proposed CARGs respond to stress in other tissues. Very recently, Cohen and Leach [27] also demonstrated that meta-analysis of diverse transcriptomic data sets in rice is a valid and robust approach to develop hypotheses for how plants respond to various stress. They discovered a list of novel candidate genes for improving rice environmental stress tolerance, which were not detected in studies focused on a single stress. Similarly, meta-analysis of transcriptome data has been performed to find out key genes in responses to abiotic stresses in other important plants [45,49,50,51,52].

The proposed sesame CARGs are functionally diversified as evidenced by the various biological pathways contributed by these genes (Figure S3). Several genes within the sesame CARGs are universally known to be abiotic stress responsive genes. For example, we detected the gene *SIN_1012768* predicted to be a member of the late embryogenesis abundant (LEA) family. Proteins encoded by the LEA are demonstrated to play defensive roles in plants during abiotic stresses, including cold, drought, salinity [53,54,55,56,57]. Other well-known abiotic stress marker genes are the alcohol dehydrogenase (ADH) family members, which are key enzymes responsible for catalyzing the reduction of acetaldehyde to ethanol using NADH as reductant, particularly during the periods of anaerobic stress [58,59]. ADH genes are involved in various environmental stresses such as drought cold, salinity, hypoxia and pathogen infection [60,61,62,63,64]. In the present study, we identified one ADH gene (*SIN_1013309*) in the sesame CARGs. Besides, we also detected several important plant abiotic stress marker genes within the sesame CARGs, including peroxidase (*SIN_1026962* and *SIN_1013457*), DREB (*SIN_1015595*), universal stress protein (*SIN_1022749* and *SIN_1012609*), glutathione *S*-transferase (*SIN_1017866* and *SIN_1002858*), 1-aminocyclopropane-1-carboxylate oxidase (*SIN_1024757*, *SIN_1007659*, *SIN_1023068*, *SIN_1009668* and *SIN_1026934*), heat shock protein (*SIN_1008669*, *SIN_1008679*, *SIN_1008672* and *SIN_1008670*) [65,66,67,68,69,70,71,72,73,74,75,76,77,78,79,80,81] etc. In future abiotic stress treatment experiments in sesame, we propose to select some of these well-known CARGs as abiotic stress marker genes in order to gauge the stress levels. Noteworthy, several uncharacterized genes were present in the CARGs, providing an exciting gene repertoire to further illuminate the complex mechanisms of plant responses to multiple abiotic stresses. 

We employed the weighted gene co-expression network analysis (WGCNA) to break down the sesame CARGs into three functional modules (Figure 2A,B). Interestingly, the functional characterization of these three modules revealed that they are involved in distinct biological pathways in response to abiotic stresses (Figure S3). With the WGCNA package, we correlated the different abiotic stressors to the gene modules (Figure 2C). This analysis is cardinal because it allowed the identification of the synergistic and antagonistic gene modules of abiotic stress response in sesame. We found that the co-expressed modules of the sesame CARGs globally displayed positive correlations with waterlogging, drought and osmotic stresses, but they were all negatively correlated with salinity stress. This suggests that manipulation of master genes of these modules to simultaneously enhance tolerance to all the four investigated abiotic stresses may not be possible in sesame, because enhancing tolerance to waterlogging, drought and osmotic stresses will lead to an increase sensitivity to salinity stress. Our findings are not surprising, since previous studies have also shown plant antagonistic responses to some stresses [9,14].

Transcription factors (TF) are regulatory molecules that play central roles in gene transcription and promote plant adaptation to various environmental conditions. The sesame CARGs contained several TFs, with ERF, MYB, bHLH and WRKY being the more predominant families (Figure 1C). A similar meta-analysis in cotton also underscored the important role of these TF families in abiotic stress responses [52], indicating a conserved abiotic stress gene regulation mechanism in plants. ERF family has been one the most studied genes in plants. Extensive studies have shown that ERF genes are essential in responses to a wide range of abiotic stresses mediated by the plant hormone ethylene [82,83,84,85,86]. It has been reported that MYB TFs also play prominent roles in triggering the right response upon exposure of plants to abiotic stresses through the ABA-dependent and independent pathways (reviewed by Li et al. [87]; Roy [88]). The WRKY genes are among the top four TF families highly active in the transcriptional reprogramming during stress and act principally through the ABA mediated pathways [73,89]. Conversely, the role and regulatory mechanisms of bHLH genes in plant abiotic stresses responses are still elusive [90]. Therefore, the sesame bHLH genes detected as key regulators of abiotic stress responsive genes in this study may represent candidate genes for the elucidation of bHLH abiotic stress regulation mechanism in plants. Overall, the diversity of TFs within the sesame CARGs highlights the complex network of interacting pathways which shape the responses to multiple abiotic stresses. To further pinpoint the master players among the large number of detected TFs, we identified in the promoter of genes within each module, the enriched putative *cis*-regulatory motifs. Previous works in yeast and human have demonstrated that genes with similar expression patterns are regulated by the same set of TFs, and therefore are likely to have similar *cis*-regulatory motifs in their upstream promoter regions [91,92]. Our study corroborated well these conclusions and unraveled for each module the master TFs that may regulate the gene expression under specific abiotic stress (Figure 4).

In gene networks, many genes only interact with a limited number of other genes, whereas a smaller subset of genes (hub genes) interacts with many other genes and therefore has a more central role [93]. Hub genes are expected to play preponderant and essential functions in organism’s fitness and according to Jeong et al. [94], hub genes are three times more likely to be essential than genes with fewer interaction partners. To test this hypothesis in our predicted gene networks (Figure 3), we selected a hub gene (*SiERF5*) and a non-hub gene (*SiNAC104*), both being transcription factors. Over-expression of these two genes in *Arabidopsis thaliana* resulted in an enhanced tolerance to drought, waterlogging and osmotic stresses, but the over-expressing lines did not tolerate salinity stress (Figure 5 and Figure 6). Furthermore, we observed that the transgenic lines over-expressing the hub gene had a stronger fitness and a higher performance under abiotic stresses compared to those transformed with the non-hub gene. It is worth mentioning that the over-expression of the hub gene had clear pleiotropic effects beyond abiotic stress responses in *Arabidopsis thaliana*, thus might play a central role in various biological pathways. This experiment therefore highlighted three key findings: (1) perturbation of a hub gene is likely to have a major fitness consequence than a non-hub gene; (2) proper manipulation of sesame CARGs may confer tolerance to multiple abiotic stresses; (3) genetic manipulation for generating sesame lines tolerant to all the four investigated abiotic stresses may be challenging due to the antagonistic response of the sesame CARGs in the face of some abiotic stresses. Although the main goal of this work was not to investigate the functional importance of hub genes versus non-hub genes, the preliminary result obtained from the Arabidopsis mutants will fuel a future study based on both sesame and Arabidopsis using over-expression and knock-out transgenic techniques and employing more hub genes and non-hub genes to comprehensively elucidate this important scientific question.

## 4. Materials and Methods 

### 4.1. RNA-Sequencing Datasets of Abiotic Stressed Sesame Samples

In order to decipher the core genome involved in abiotic stress responses in plants, our group previously generated several RNA-Seq data of sesame under waterlogging [6], drought [7], salt [8] and details of the experimental procedures could be found in the respective articles. In this study, we collected the root RNA-Seq data of the waterlogging-tolerant genotype Zhongzhi13 under 3 h waterlogging stress and control condition (SRR2886790). Concerning the drought stress, we collected the root RNA-Seq data from a drought-tolerant genotype ZZM0635 (SAMN06130606) and a drought-sensitive genotype ZZM4782 (SAMN06130607) after 0, 3, 7 and 11 days drought stress. For the salt stress experiment, two contrasting genotypes (salt-tolerant WZM3063 and salt-sensitive ZZM4028) were treated with 150mM NaCl and whole seedling samples were collected at 0, 2, 6, 12 and 24 h (PRJNA524278). In addition to these released datasets, we newly generated an RNA-Seq data from root samples of osmotic stressed sesame (PRJNA552167). Two sesame genotypes (osmotic-tolerant G546 and osmotic-sensitive G259) grown in a box containing 9 L of half-strength Hoagland solution for 21 days, were treated with 2% PEG6000 for 7 days. Samples were collected in triplicate from stress and control conditions after the stress period, immediately placed in liquid nitrogen and stored at −80 °C until use. 

### 4.2. Total RNA Isolation and Sequencing from the PEG6000-Treated Seedlings

Total RNA of the 12 PEG6000-treated samples was extracted using an EASYspin Plus kit (Aidlab, Beijing, China). The cDNA libraries generated from RNA samples were pair-end sequenced on an Illumina Hiseq 4000 platform (San Diego, California, CA, USA.) according to the methods described by Dossa et al. [7].

### 4.3. RNA-Seq Data Analysis

A total of 72 RNA-Seq data, including 30, 24, 6 and 12 data from salt, drought, waterlogging and osmotic stresses, respectively, were used in this study. The raw data were first processed with FastQC (http://www.bioinformatics.babraham.ac.uk/projects/fastqc/) to filter out adapters and low-quality sequences. The raw data was submitted to NCBI SRA (PRJNA552167). Then, the clean reads were mapped to the sesame genome v.1.0 (https://www.ncbi.nlm.nih.gov/genome/?term=sesamum) [95] using HISAT [96]. The RSEM package v1.3.0 [97] was used to calculate gene expression level for each sample expressed as fragments per kilobase of transcript per million fragments mapped (FPKM). For each treatment, the gene expression levels in the stressed samples were compared with those in the control samples in order to identify the differentially expressed genes (DEG). The DEGs were detected as described by Tarazona et al. [98] based on the parameters: Fold change ≥ 2 and Probability ≥ 0.8, with a significant false discovery rate-adjusted *p* value (FDR) < 0.05 based on the three biological replicates. Gene Ontology enrichment analysis for the DEGs was performed using the clusterProfiler version 3.8. 

### 4.4. Analysis of Co-Expression Modules Based on WGCNA

Weighted Gene Co-Expression Network Analysis (WGCNA) package version: 1.61 [39] in the R software (http://www.r-project.org/) was used to construct the gene co-expression networks from the normalized log2-transformed FPKM matrix as described by Wan et al. [99] and Yang et al. [100]. This analysis was based on the core conserved DEGs between the four abiotic stress treatments. The gradient method was used to test the independence and the average connectivity degree of different modules with different power value (the power value ranging from 1 to 20). The appropriate power value was determined when the degree of independence was 0.8. Then, the adjacency was transformed into a topological overlap matrix (TOM), which could measure the network connectivity of a gene defined as the sum of its adjacency with all other genes for network generation, and the corresponding dissimilarity (1-TOM) was calculated. To classify genes with similar expression profiles into gene modules, average linkage hierarchical clustering was conducted according to the TOM-based dissimilarity measure with a minimum size (gene group) of 50 for the genes’ dendrogram.

Module-trait associations were estimated using the correlation between the module eigengene and the stress treatments. Network visualization for each module was performed using the Cytoscape software version 3.6.1 [101] with a cut off of the weight parameter (obtained from the WGCNA) set at 0.30.

### 4.5. Enrichment Analysis of Cis-Regulatory Motifs 

To detect the enriched *cis*-regulatory motifs within the promoters of the genes belonging to each module detected by WGCNA, first, all the sesame transcription factor binding motifs were downloaded from the JASPAR website (http://jaspar.genereg.net/, [102]. Then, the sequences of 1 kb upstream from the transcription start sites of the genes belonging to each module detected by WGCNA were retrieved from the sesame genome v1.0 [95]. The promoter regions were scanned for presence/absence of the DNA binding motifs using the FIMO tool v5.0.3 [103] with a threshold (*p* < 0.01). Finally, we performed an enrichment analysis of each motif within the promoters of the genes belonging to each module using the cumulative hypergeometric distribution (*p* ≤ 0.05). 

### 4.6. Vector Construction and Arabidopsis Genetic Transformation

We selected two genes, including a hub gene (*SiERF5*, *SIN_1005239*) and a no-hub gene (*SiNAC104*, *SIN_1026079*) to demonstrate their involvement in abiotic stress responses in *Arabidopsis thaliana* following descriptions of Dossa et al. [38]. Briefly, the protein coding region were cloned by PCR from sesame root cDNA (SiERF5-F-GCTTTCGCGAGCTCGGTACCATGAGAATGATTCTCAAGAA, SiERF5-R-CGACTCTAGAGGATCCTGTCAAGTGAGATGGTTTGA); (SiNAC104-F- GCTTTCGCGAGCTCGGTACCATGGCTGAAGGGAGGAAATG, SiNAC104-R- CGACTCTAGAGGATCCAGATCAGCTTGCCTAACTAG) and inserted into a pCAMBIA 1301s vector (which is a modified form of the pCAMBIA1301 vector) between KPnI (5′-end) and BamHI (3′-end) sites, driven by the CaMV 35S promoter. The plasmids containing the 35S::SiERF5 and 35S::SiNAC104 constructs were transformed first into *Agrobacterium tumefaciens* strain LBA4404 and then into Arabidopsis ecotype Col-0 cv. Columbia by the floral dipping method [104]. Transgenic seeds were screened by sowing on MS medium containing 1% agar, and 1% sucrose and 50 μg·ml^−1^ hygromicin. All the putative T1 transgenic plants and vector control (VC) plants (containing an empty pCAMBIA1301 vector) were screened by PCR with genomic DNA from leaves. Furthermore, qRT-PCR was performed to confirm the expression of the transgene [105]. Three independent T3 transgenic homozygous lines were used for the stress treatments, gene expression assay and phenotypic analyses.

### 4.7. Evaluation of Transgenic Lines Exposed to Osmotic, Salt, Waterlogging and Drought Stresses

First, to analyze the response of the transgenic plants to osmotic stress, seeds of VC and three T3 lines for each transgene, were surface sterilized and plated on solid MS medium. The seeds were stratified for 2 days in the dark at 4 °C and then transferred to a growth chamber under a 16-h light period (long-day condition) provided by fluorescent light at 120 µM·m^−2^·s^−1^ and day/night temperatures of 22/16 °C and 60/75% relative humidity. 10 days-old seedlings were transferred into solid MS medium supplemented with 0/250 mM Mannitol. Plates were placed vertically and after 10 days, seedling root length was recorded. 

Next, 10-day-old seedlings (transgenic lines and VC plants) were transferred into pots (two plants per pot) containing organic potting mix and grown in normal conditions for 15 days. Then, 1/4 of the pots were subjected to dehydration stress by withholding watering for 20 days and subsequently, plants were allowed to recover for 1 week by supplementing water [38]. Another 1/4 of the pots were watered with 200 mM NaCl solution every three days for four times and subsequently, plants were allowed to recover for 1 week by supplementing water [38]. Another 1/4 of the pots were subjected to waterlogging stress for 18 days. Pots were placed inside plastic tanks and filled with tap water up to 5 mm above the ground [106]. After waterlogging stress, plants were allowed to recover for 1 week by drainage. The remaining plants (1/4) were kept under normal growth condition throughout the experiment. Leaf samples were collected at the end of each stress treatment and in the control condition for gene expression analysis. After recovery, the plant survival rate, the above-ground rosette biomass fresh weights were recorded and pictures were captured to show visible phenotypes. We estimated the relative rosette biomass as the ratio of the records under stress and control conditions. For each treatment, eight survived plants (four pots) were kept until maturation to evaluate the seed yield. The experiment was repeated twice with four replicates in each experiment for statistical analysis.

### 4.8. Sesame Materials and Stress Treatments

Ten cultivars of sesame were obtained from the China National Genebank, Oil Crops Research Institute, Chinese Academy of Agricultural Sciences and used in this experiment. The genotypes G059, G079, G207, G208, G209, G210, G212, G213, G214 and G215, all originating in China, were used in this experiment. The sesame seeds were sterilized with 3% sodium hypochlorite for 7 min and washed three times using sterile water. For the drought experiment, the seeds were sown in pots containing loam soil mixed with 10% vermiculite and plants were regularly watered. After 6 weeks, seedlings were submitted to a water stress for 7 days [7]. For the waterlogging treatment, seedlings were flooded by standing in a plastic bucket filled with tap water to 3 cm above the soil surface and maintained for 9 h according to the experimental descriptions of Dossa et al. [105]. Concerning the salt, heat and osmotic stress treatments, seedlings were hydroponically grown in a box containing half-strength Hoagland solution for 2 weeks under ambient temperature of 35 °C. Then, they were transferred to a new nutrient solution containing 200 mM NaCl for 48 h (salt stress treatment), in a nutrient solution containing 2% PEG6000 for 5 days (osmotic stress treatment) or under 45 °C for 48 h (heat stress treatment). Root samples of stressed and control plants were collected at the same periods.

### 4.9. Gene Expression Analysis in Arabidopsis

The qRT-PCR was performed on RNA extracted from leaf samples (Arabidopsis) and root samples (sesame). The genes *Actin 2* (*AT3G18780*) and *Actin 7* (*SIN_1006268*) were used as the internal control for Arabidopsis and sesame, respectively. Specific primer pairs of the assayed genes were designed using the Primer5.0 software [107] (Table S1). Samples in the control condition (non-stress) were used as reference and data are presented as relative transcript level based on the 2^−∆∆*C*t^ method [108].

### 4.10. Statistical Analysis

All the data were analyzed with the R software (www.r-project.org) using the one-way analysis of variance for significant difference statistical analysis. The error bars were calculated with data from two independent experiments.

## 5. Conclusions

Using the meta-analysis approach coupled with the weighted gene co-expression network analysis on 72 RNA-Seq datasets from drought, salt, osmotic and waterlogging treatments, we decoded the core abiotic stress responsive genes (CARG) in sesame. In total, 543 genes were detected as the sesame CARGs, some of which were experimentally validated. The CARGs were further divided into three distinct functional modules, which are involved a wide range of biological pathways. Module-traits association analysis provided insights into the synergistic and antagonistic gene modules of abiotic stress response mechanisms in sesame. The stress specific expression patterns of genes within the different modules are tightly regulated by key transcription factors from the families of ERF, WRKY, MYB and bHLH. Moreover, a set of hub genes and master regulators predicted to play prominent functions for abiotic stresses responses in sesame was identified, representing useful resources of molecular biomarkers and highly-anticipated potential candidate genes for engineering multiple stresses tolerance in sesame.

## Figures and Tables

**Table 1:** Characteristics of the RNA-Seq datasets used in this study.

Characteristics	Salt	Drought	Waterlogging	Osmotic	Total
Number of samples	30	24	6	12	72
Clean reads (Gb)	230	160	13.5	83.1	486.6
Total expressed genes	23,415	24,113	21,064	22,418	25,319

## Supplementary Materials

Supplementary materials can be found at https://www.mdpi.com/1422-0067/20/16/3930/s1.
