# Supplementary material for: Depicting the Core Transcriptome Modulating Multiple Abiotic Stresses Responses in Sesame (Sesamum indicum L.)
Source: Int J Mol Sci. 2019 Aug 13;20(16):3930. doi: 10.3390/ijms20163930 (PMC6721054; doi:10.3390/ijms20163930)
Supplement: Supplementary file 1 [file ijms-20-03930-s001.zip › ijms-557269 - supplementary .docx]

**Figure S1.** Heatmap displaying the expression value (FPKM) of the 543 genes representing the core abiotic-stress responsive genes in sesame. ST = salt tolerant genotype, SS = salt sensitive genotype, WT = waterlogging tolerant genotype, DS = drought sensitive genotype, DT = drought tolerant genotype, OS = osmotic sensitive genotype, OT = osmotic tolerant genotype.

(The picture is very large, please read the PDF version of Figure S1 in the supplementary materials)


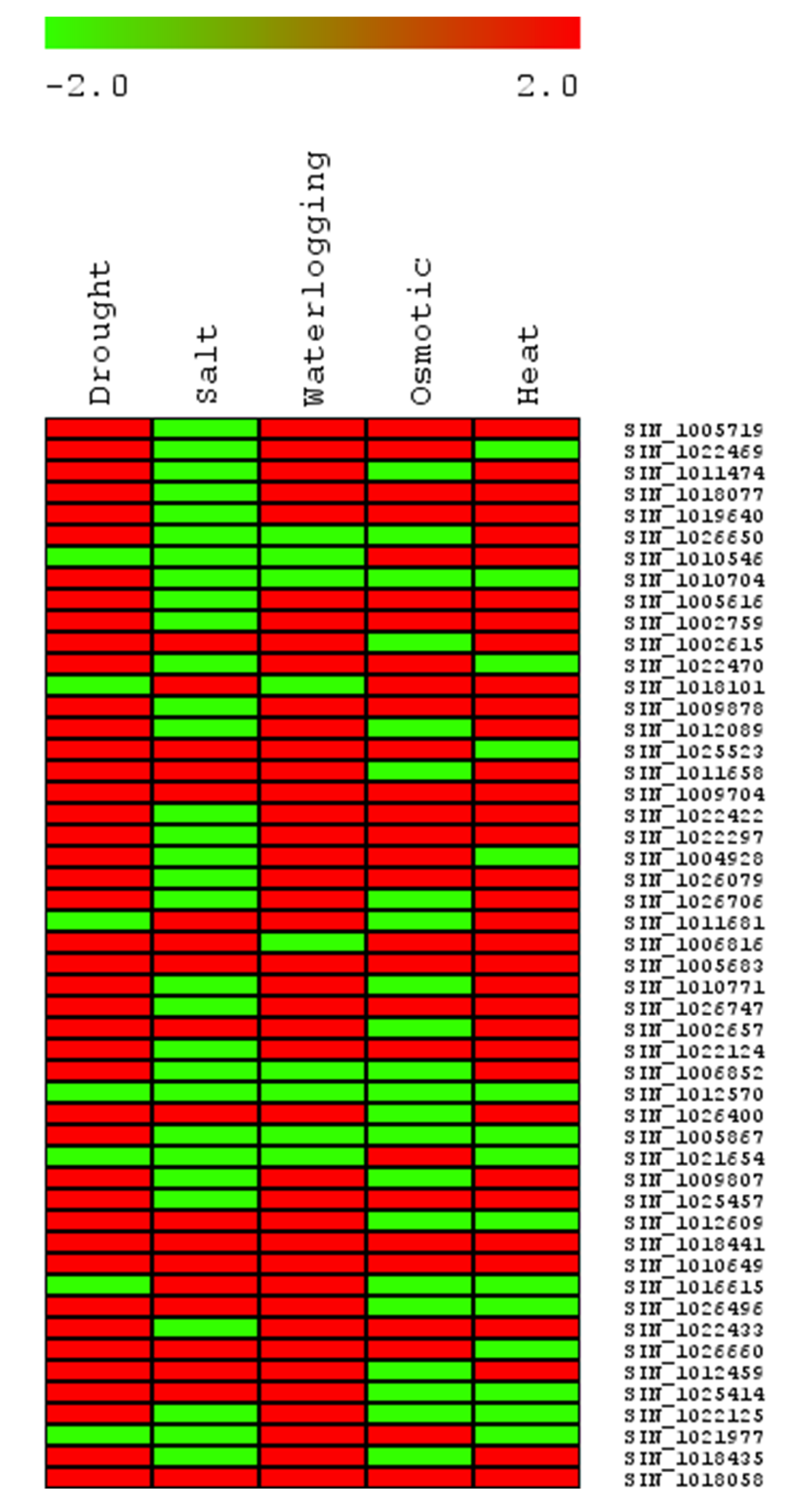


**Figure S2.** Heatmap displaying the average log2 fold change of the expression value of 50 selected genes from the sesame core abiotic stress responsive genes (CARG) in 10 sesame genotypes under drought, NaCl, waterlogging and heat stresses. Green color means down-regulation, while red color means up-regulation.

**Figure S3.** GO enrichment analysis of the co-expressed gene modules. (**A**) GO of Blue module. (**B**) GO enrichment analysis of Grey module. (**C**) GO enrichment analysis of Turquoise module.


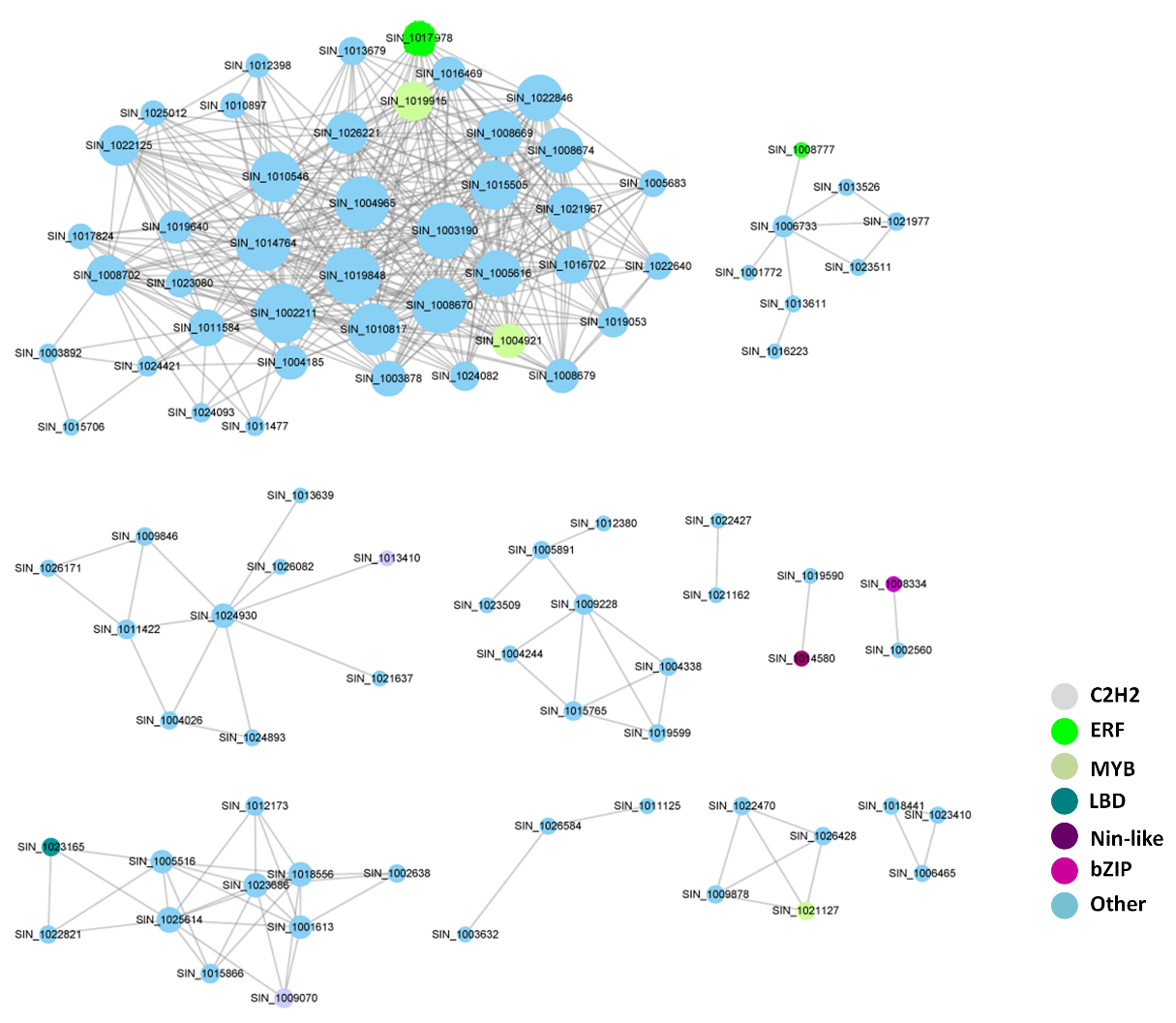


**Figure S4.** Network topology of Grey module detected by WGCNA. The size of node circle is positively correlated with the number of the interacting gene partners.


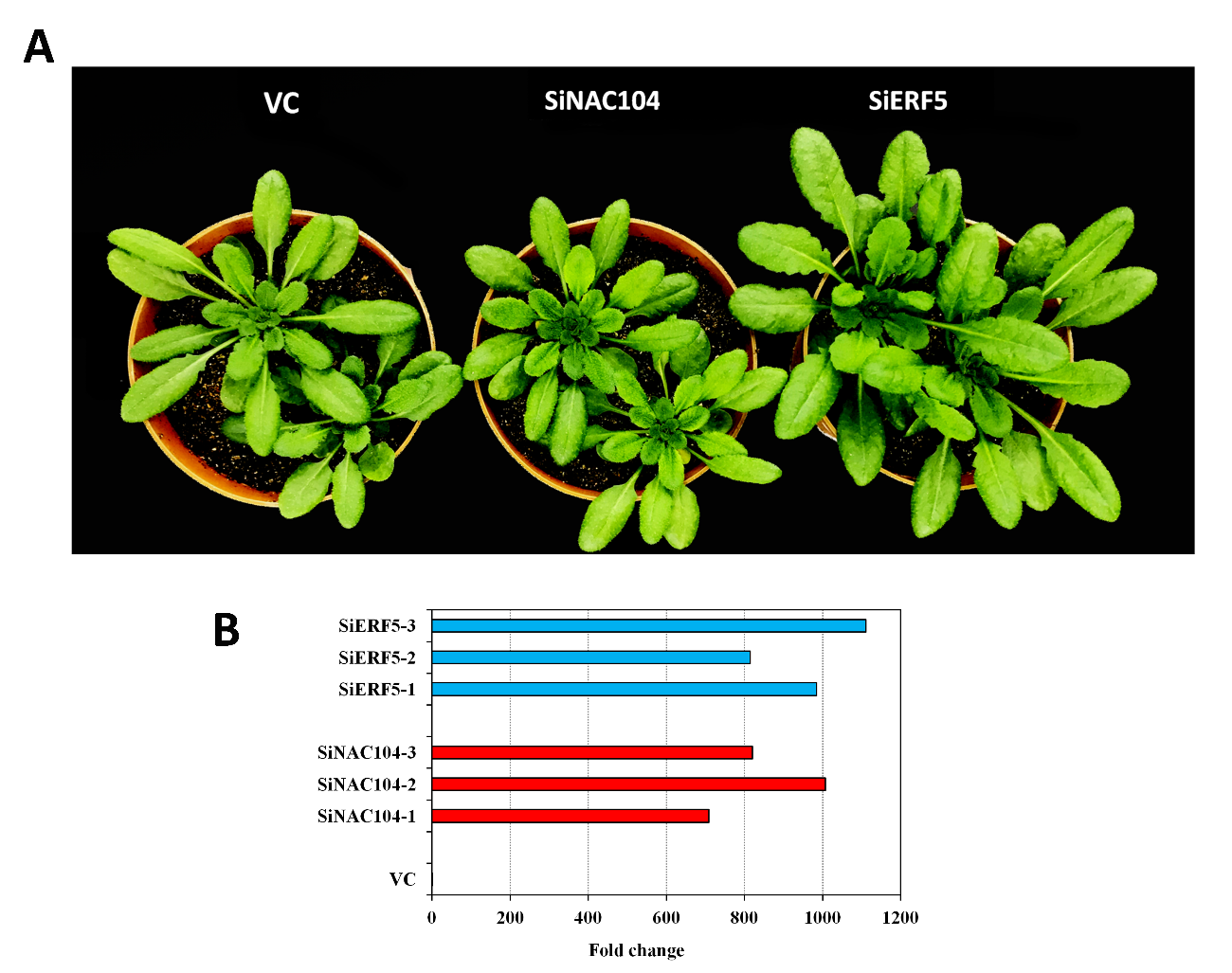


**Figure S5.** Generation of overexpressing *Arabidopsis thaliana* lines carrying the genes *SiERF5* and *SiNAC104*. (**A**) Effect of the transgenes on the plant growth and development. (**B**) qRT-PCR validation of the expression of the transgenes in three selected transgenic lines in the T3 generation. VC = vector control.

Table title:

**Table S1.** List of the primers used for qRT-PCR experiments in sesame and *Arabidopsis thaliana*.

**Table S2**. Expression value (FPKM) of the 543 genes representing the core abiotic-stress responsive genes in sesame and their predicted function. ST = salt tolerant genotype, SS = salt sensitive genotype, WT = waterlogging tolerant genotype, DS = drought sensitive genotype, DT = drought tolerant genotype, OS = osmotic sensitive genotype, OT = osmotic tolerant genotype.

**Table S3.** Repartition of the sesame core abiotic-stress responsive genes into the three co-expressed modules (Grey, Blue and Turquoise) detected by WGCNA.

**Table S4**. Identification of the enriched cis-regulatory motifs in the promoters of the genes in each co-expressed module detected by WGCNA. The cumulative hypergeometric distribution (*p* ≤ 0.05) was used to identify the enriched motifs. For each motif, the corresponding transcription factor was supplied together with the predicted target genes
