# Supplementary figures and images for: Depicting the Core Transcriptome Modulating Multiple Abiotic Stresses Responses in Sesame (Sesamum indicum L.)
Source: Int J Mol Sci. 2019 Aug 13;20(16):3930. doi: 10.3390/ijms20163930 (PMC6721054; doi:10.3390/ijms20163930)

sbheatmap

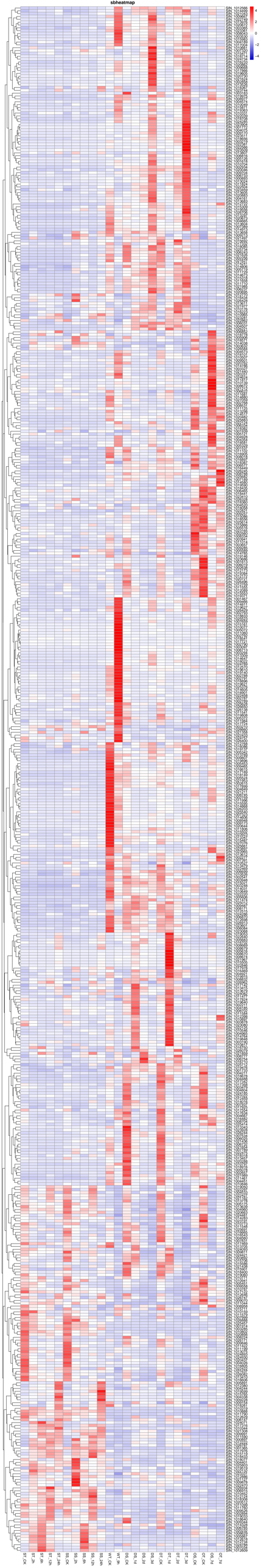

Supplement: Supplementary file 1 [file ijms-20-03930-s001.zip › Figure S1.pdf]
